# Cannabinoids and the endocannabinoid system in the regulation of cytochrome P450 metabolic activity-a review

**DOI:** 10.3389/fphar.2025.1599012

**Published:** 2025-06-05

**Authors:** Carlos D.F. Fonseca, Ondřej Zendulka, Jan Juřica

**Affiliations:** ^1^ Department of Pharmacology, Faculty of Medicine, Masaryk University, Brno, Czechia; ^2^ Masaryk Memorial Cancer Institute, Brno, Czechia; ^3^ Department of Pharmacology and Toxicology, Faculty of Pharmacy, Masaryk University, Brno, Czechia

**Keywords:** cannabinoids, endocannabinoid system, cytochrome P450, metabolism, hypothalamic-pituitary axis, cannabinoid-based therapies

## Abstract

The use of cannabinoids has a history spanning thousands of years, and their pharmacological and toxicological properties, particularly those of THC and CBD, are well-documented. However, their potential to induce drug-drug interactions remains underexplored. This review aims to provide a comprehensive perspective by contextualizing the historical and pharmacological significance of cannabinoids while focusing on their capacity to modulate the metabolic activity of cytochrome P450 isoforms relevant to drug metabolism. Additionally, we look at the impact of cannabinoids in neuronal circuits impacting the hypothalamic-pituitary hormonal axis, such as the *locus coeruleus* and *raphe nuclei* and their possible consequences on the cytochrome P450 system. Recognising potential interactions between cannabinoids and other drugs could enhance understanding of their pharmacological effects, improve the efficacy and safety profiles of cannabinoid-based therapies, and encourage further exploration into this under-researched area of psychopharmacology, with implications for both preclinical research and clinical practice.

## 1 Background

Cannabis has been used for its medicinal effects for almost five thousand years - the first mention comes from China and dates back to 2737 BC (Zuardi, 2006). Importance was predominantly placed on the nutritional value of the seeds within these regions. In ancient and medieval cultures, it was predominantly used (in addition to its psychoactive effect) to treat a variety of somatic diseases, including headache, fever, bacterial infections, diarrhea, rheumatic pain, and malaria. The 19th century brought extensive medical literature around the use of cannabis, with one of the most notable works being *The Hasheesh Eater* (1857) of Fitz Hugh Ludlow. *The Emperor Wears No Clothes: The Authoritative Historical Record of Cannabis and the Conspiracy Against Marijuana* of Jack Herer demonstrates the hysteria around the use of cannabis during this period in the United States of America (USA) “… from 1842 until the end of the 19th century, marijuana, … was one of three substances (after alcohol and opium) more commonly used (in massive doses, generally by oral ingestion)”. The end of the 19th century brought decline and replacement by opium derivates easier to use (Menezes, 2024). A renaissance of its use followed at the beginning of the 20th century with personalities like Queen Victoria and Empress Sissi, who used it for its antitussive properties and appetite stimulation (Crocq, 2020). Protocols for the preparation of extracts and tinctures were incorporated in the third edition of the American Pharmacopoeia with the intention of treatment of mental disorders. However, they were later removed due to the introduction of the Marihuana Tax Act of 1937 in the USA (Aggarwal et al., 2009). Throughout most of the 20th century, cannabis use for medical purposes was limited due to a lack of knowledge about its active substance, (−)-trans-Δ^9^-tetrahydrocannabinol (THC), which was discovered in 1964 (Gaoni and Mechoulam, 1964), followed by the discovery of cannabinoid receptors and in 1992 also the discovery of endocannabinoids (Devane et al., 1992).

There is an ongoing debate among the botanical community as to whether cannabis exists as a single species (*Cannabis sativa* with different subspecies and varieties) or whether there are three separate species: *Cannabis sativa*, *Cannabis indica*, and *Cannabis ruderalis*. The nomenclature of the plant is based on the organoleptic properties of the plant, the cannabinoid and terpenoid content, and even the habitus of the plant itself, like the shape or size of leaves (Grotenhermen and Russo, 2002). Cannabinoids are a group of compounds present in the plant of cannabis directly alongside others like terpenoids. Grotenhermen and Russo (2002) write that the best-known cannabinoids are THC and cannabidiol (CBD), and depending on the plant, the concentration and ratio can vary.

## 2 Pharmacology of the cannabinoids and the endocannabinoid system

### 2.1 Cannabinoids

The group of cannabinoids comprise phytocannabinoids, endocannabinoids, and synthetic cannabinoids. Phytocannabinoids are all cannabinoids isolated from *Cannabis sativa*, *Cannabis indica* and *Cannabis ruderalis*. Endocannabinoids are molecules produced in the human body and fit into different chemical classes (Grotenhermen and Russo, 2002). Synthetic cannabinoids are chemically promiscuous compounds (Roque-Bravo et al., 2023), which we will delve deeper into further. Besides the two best-known phytocannabinoids, THC and CBD over 120 other phytocannabinoids were identified. Monoterpenoids (e.g., myrcene, α-pinene, and limonene) have significant pharmacological effects through the direct activation of the CB_1_ receptor with variable amplitude response (between 10% and 48%) of 10 µM THC (Raz et al., 2023). Besides the direct interaction with CB_1_, Raz et al. (2023) determined that monoterpenoids can augment THC-mediated activation of CB_1_ receptor which, depending on the type of monoterpenoid, can be the result of a summation of effects (e.g., linalool) or potentiation (e.g., limonene).

THC was isolated in 1964 by Raphael Mechoulam and colleagues (Gaoni and Mechoulam, 1964). THC can interact as a partial agonist on cannabinoid receptors (Pertwee, 2008). There are two types of cannabinoid receptors: CB_1_ and CB_2_. These are G-protein coupled receptors and have different prevalences in the human body (Nyíri et al., 2005). While CB_1_ receptors are more readily found in the central nervous system (CNS), the CB_2_ receptors are in microglia, osteoclasts, and macrophages (Grotenhermen and Russo, 2002; Nyíri et al., 2005; Atakan, 2012; Groce, 2018). The psychoactive properties of THC can be attributed to the partial agonism of CB_1_ receptors. This interaction causes symptoms like drowsiness, increased appetite, or short-term memory loss (Grotenhermen and Russo, 2002). Reducing the effects of THC merely to its “psychoactive” purposes would be wrong and an understatement. Analgesic, antipruritic, antiemetic, neuroprotective, and bronchodilatory properties have been described so far (Groce, 2018). The effects of THC should not be separated from that of CBD because CBD is a negative allosteric modulator of CB_1_ (Laprairie et al., 2015). Unlike THC, it does not act directly on the orthosteric site but allosterically decreases the efficacy of orthosteric ligands such as THC. It is believed that it has a neuroprotective effect while avoiding the intoxication caused by THC. This detail can be the main reason why CBD is claimed to be a non-psychoactive molecule, even though it has psychoactive (modulatory) actions (Russo, 2017). Anxiolytic action (Resstel et al., 2009) and anti-convulsant role (Carlini and Cunha, 1981) have also been described. This anxiolytic action possibly results from the agonism of the 5-HT_1A_ receptor (Resstel et al., 2009).

Cannabigerol (CBG) is another non-psychoactive phytocannabinoid found in *Cannabis sativa*. Like THC, CBG was synthesized and isolated in 1964 (Gaoni and Mechoulam, 1964); however, its clinical significance and research attention have remained relatively limited compared to THC and CBD. The latter compounds have been incorporated into clinical practice, notably in the form of oromucosal spray *Sativex* ^®^ (GW Pharma Ltd.), a registered medicine in the European Union used to alleviate symptoms of multiple sclerosis. In contrast to these more extensively studied phytocannabinoids, CBG exhibits negligible activity at the CB_1_ receptor and acts as a partial agonist at CB_2_ (Navarro et al., 2018). Notably, CBG is believed to be a potent agonist of the α_2_-adrenoceptor at nanomolar concentrations, suggesting potential relevance in the development of antihypertensive therapies and in the treatment of psychiatric conditions such as post-traumatic stress disorder and attention-deficit disorder. Additionally, CBG demonstrates strong antagonistic activity at the 5-HT_1A_ receptor (Cascio et al., 2010). CBG also causes the activation of the PPARγ receptor (Atalay et al., 2019), which contributes to reducing inflammation (Granja et al., 2012).

### 2.2 Position of the regulatory agencies to the implementation of cannabinoids in clinical use

The integration of cannabinoids into clinical practice is tightly regulated and must meet the same core regulatory requirements related to safety, efficacy, quality, and manufacturing standards as any other drug. However, in practice, cannabinoid-based drugs often face additional scrutiny due to their association with controlled substances and the legal status of cannabis under national and international drug control laws. In the United States, the Food and Drug Administration (FDA) has approved only a few cannabinoid-based medications, such as *Epidiolex* ^®^ (Greenwich Biosciences) for specific seizure disorders, and synthetic cannabinoids like dronabinol for chemotherapy-induced nausea (U.S. Food and Drug Administration, 2020). In the European Union, the European Medicines Agency (EMA) follows similar principles, requiring comprehensive clinical data and adherence to Good Manufacturing Practice (GMP) (European Medicines Agency, 2019). Both agencies emphasize the need for well-controlled studies, pharmacovigilance plans, and clear evidence of benefit over risk. Besides these clinically used drugs, crude dried female flowers of Cannabis sativa or Cannabis indica, known as “medical cannabis,” have been approved in some countries (e.g., the UK, Germany, France—pilot use only, Netherlands, Switzerland, Denmark, Italy, Portugal, Finland, Norway, Poland, and the Czech Republic), while in others it is still prohibited, highly restricted, or approved only in clinical trials (e.g., Slovakia, Bulgaria, Serbia, Hungary, Latvia). Legislation and rules about prescription and reimbursement in different countries were recently revised elsewhere (Baratta et al., 2022). The concentration of active constituents in medical cannabis must be precisely specified, with THC and CBD content typically ranging from approximately 0.1%–20%, as determined by accredited laboratory analysis. Medical cannabis may be clinically used in several indications, including chronic pain (particularly cancer-related pain), neuropathic pain, glaucoma-associated pain, spasticity and spasticity-related pain in multiple sclerosis or spinal cord injury, dyskinesias, and other complications caused by neurological disorders or injuries to the spine or brain, including Parkinsonian tremor (U.S. Food and Drug Administration, 2023; Landa et al., 2018).

### 2.3 Synthetic cannabinoids

Besides phytocannabinoids, synthetic cannabinoids (SCs) are part of NPS (new psychoactive substances). Roque-Bravo and colleagues (Roque-Bravo et al., 2023) provided an extensive review of these substances. There are numerous SCs with various structures, often non-related to phytocannabinoids or endocannabinoids, and they can be incorporated into different chemical classes: aminoalkylindoles (e.g., WIN55), indazole carboxamides, naphthoylindoles (e.g., JWH-015), pyrazole derivatives (e.g., AM-251) and many more (Suriaga et al., 2023). SCs are lipophilic substances mainly inhaled and rarely consumed by herbal infusions as a tea. From the pharmacological point of view, even taking into consideration structural differences most of the SCs are full agonists of CB_1_ and CB_2_, which activation causes a decrease in adenyl cyclase activity and, consequently, a decrease in cAMP and protein kinase A (PKA) in the presynaptic neuron. The activation also contributes to the inhibition of influx of Ca^2+^ and stimulation of efflux of K^+^ which hyperpolarizes the membrane and makes the release of neurotransmitters impossible. In the postsynaptic neuron, the binding of SCs to CB_1_ and CB_2_ contributes to the activation of kinases such as mitogen-activated protein kinase (MAPK) and extracellular kinases 1 and 2 (ERK1/2). The binding to orphan receptors (e.g., GPR55) leads to increased intracellular Ca^2+^ concentrations. Interaction with PPAR- γ nuclear receptor leads to regulation of gene transcription (Roque-Bravo et al., 2023). Various partial agonists, antagonists or inverse agonists were also synthesized and studied by Roque-Bravo et al. (2023).

### 2.4 Endocannabinoids and endocannabinoid system

Endocannabinoids (ECs) are endogenous ligands for cannabinoid receptors. These lipophilic compounds are eicosanoids derived from arachidonic acid. Anandamide (N-arachidonoylethanolamine) and 2-arachidonoyglycerol (2-AG) were the first ECs discovered in 1992 (Devane et al., 1992) and 1995 (Mechoulam et al., 1995). They are an integral part of the endocannabinoid system and act as retrograde regulators of glutamate, gamma-aminobutyric acid (GABA), acetylcholine, and serotonin (Katzung, 2018). Anandamide is a partial agonist (with higher affinity than 2-AG) of cannabinoid receptors but can also bind to other kind of receptors (e.g., TRPV1 and TRPV4) (Pertwee, 2008). 2-AG is a selective full agonist of cannabinoid receptors (Gonsiorek et al., 2000). Other endocannabinoids, such as virodhamine and oleamide, have a chemical structure similar to anandamide, while noladin ether is similar to 2-AG (Rodríguez de Fonseca et al., 2005).

Anandamide is biosynthesized from N-acylphosphatidylethanolamines (NAPEs) through four pathways. The most important is the classic pathway, where the hydrolysis of NAPEs occurs by NAPE-specific phospholipase D (NAPE-PLD). 2-AG is the product of the action of phospholipase-C, phosphatases, and lipases α/β that catalyze the breakdown of diacylglycerols (DAGs). The two main enzymes responsible for the degradation of these endocannabinoids are fatty acid amide hydrolase-1 (FAAH-1) and monoacylglycerol lipase (MAGL). FAAH-1 is responsible for the degradation of anandamide to arachidonate and ethanolamine, while MAGL is responsible for the degradation of 2-AG to arachidonate and glycerol (Deutsch and Chin, 1993; Arreaza et al., 1997). In the metabolism of anandamide, other enzymes such as FAAH-2 and N-acylethanolamine acid amidase (NAAA) also play an active role. In the metabolism of 2-AG, the MAGL and other serine hydrolases make up almost the full extent of the degradation (Evagorou et al., 2010). Interestingly, to a certain extent, FAAH-1, through condensation of arachidonate and ethanolamine, can contribute to the biosynthesis of anandamide, but the importance relative to degradation is negligible. Cyclooxygenase-2 (COX-2) metabolism of anandamide and 2-AG also occurs. This leads to the synthesis of prostaglandin-ethanolamides (or prostamides) and prostaglandins-glycerol, respectively (Simard et al., 2022). Prostamides, although agonists of CB_1_ and CB_2,_ possess conformational restraints that do not allow for high-affinity binding when compared to anandamide (Berglund et al., 1999). Prostamides, specifically prostamide F_2a_, have weak activity towards TRPV1 (Matias et al., 2004). A noticeable decrease in the production of interleukin-2 was also reported through PPARγ activation by an unknown anandamide metabolite produced by COX-2 (Rockwell and Kaminski, 2004). Other metabolic pathways of ECs, such as cytochrome P450 and 12/15-lipoxygenases (12/15-LOX), also occur (Simard et al., 2022). More recently, the term “endocannabinoidome” has been coined to describe this group of endocannabinoids, receptors, and biosynthetic and degradation enzymes (Di Marzo, 2018). Modulating the endocannabinoid system involves regulating many physiological functions in the human body, including neurobehavioral processes, hormonal regulation, metabolic pathways, and the proper functioning of the gastrointestinal tract. (Grotenhermen and Russo, 2002; Groce, 2018).

## 3 The regulation of cytochrome P450 metabolic activity

### 3.1 Induction and inhibition

Cytochrome P450 (P450) family comprise 18 families and over 50 isoenzymes (Nebert et al., 2013). Besides the substantial influence of genetic polymorphisms, the activity of P450 can be modulated by induction or inhibition. These effects may be mediated by drugs, food supplements, components of the diet, cigarette smoke, herbal compounds and hormones. Induction occurs through the interaction of an inducer with nuclear receptors (sometimes referred to as xenosensors), such as aryl hydrocarbon receptor (AhR), constitutive androstane receptor (CAR), pregnane X receptor (PXR) and the peroxisome proliferator receptors (PPAR) (Knight et al., 2008) or glucocorticoid receptor (Dvorak and Pavek, 2010). Once the interaction occurs, the xenosensors will interfere with P450 gene expression. Commonly, one of the nuclear receptors interferes with another in a process called cross-talk. Understanding the cross-talk between the major nuclear receptors and others, such as NF-κB, is also relevant for understanding the inflammation processes in the human body (Casarett et al., 2008; Zendulka et al., 2016). Inflammation due to the cross-talk between PXR and NF-κB also influences the P450 protein. Inflammation is a trigger for decreased P450 expression and drug metabolization. Inflammatory mediators (such as cytokines and interleukins) activate NF-κB, which inhibits the induction of P450 isoforms (as CYP3A4, CYP2B6, and CYP2C9) by the nuclear receptor PXR (Pavek, 2016; Lenoir et al., 2021). Unlike enzyme induction, which requires multiple doses of the interacting drug to develop, enzyme inhibition occurs immediately after the first administration of the inhibiting drug, as it directly blocks the enzyme’s activity without needing time for upregulation or synthesis. There are two types of inhibition of P450 enzymes: reversible and irreversible, the latter of which is also known as mechanism-based inhibition (Pelkonen et al., 2008). In reversible inhibition, competitive inhibition occurs between the substrate and inhibitor for the enzyme’s active site. The binding is weak, the action is rapid, and it does not inactivate the enzyme. Reversible inhibition can be further divided into uncompetitive inhibition, which occurs when the inhibitor binds to the enzyme-substrate complex, and mixed-inhibition, when both competitive and uncompetitive occur at the same time (Lin and Lu, 1998). Compared to the latter, irreversible or mechanism-based inhibition is of longer duration due to the formation of strong covalent bonds and is dependent on the formation of inhibitor metabolites. This type of binding can only be reverted by the synthesis of a new enzyme, and in some circumstances, this may not be possible due to total enzyme inactivation (Halpert, 1995).

### 3.2 Cannabinoids as regulators of cytochrome P450 metabolic activity

Yamaori and colleagues demonstrated through multiple studies using human liver microsomes and recombinant P450: CYP1A1, CYP1A2, CYP1B1 (Yamaori et al., 2010), CYP2A6, CYP2B6 (Yamaori et al., 2011a), CYP2C9 (Yamaori et al., 2012), and CYP2D6 (Yamaori et al., 2011b) that THC and its metabolites are reversible inhibitors of several isoforms of P450. The inhibition potencies of the main cannabinoids (THC, CBD, and CBN) are summarized in Table 1 below:

**TABLE 1:** The IC_50_ (µM) of the major cannabinoids investigated in recombinant human CYP enzymes (Yamaori et al., 2010; Yamaori et al., 2011a; Yamaori et al., 2011b; Yamaori et al., 2012).

	CYP1A1*	CYP1A2*	CYP1B1*	CYP2A6*	CYP2B6*	CYP2C9*		CYP2D6*
THC	ND	ND	ND	32,50	7,37	1,02	2,84	17,10
CBD	0,54	ND	ND	43,50	2,51	2,70	2,67	6,01
CBN	ND	0,19	0,28	30,40	5,65	1,13	2,86	12,30
	7-ERO-DET			COU-7-HD	7-BRO-O-DBZ	DICLO4′-HD	S-WAR7-HD	DEXO-DMT

IC_50_, half-maximal inhibitory concentration; ND, represent non-shared data; 7-ERO-DET, seven-ethoxyresorufin O-deethylase; COU-7-HD, Coumarin-7-hydroxylase; 7-BRO-O-DBZ, 7-benzoxyresorufin O-debenzylation; DICLO4′-HD, Diclofenac 4′-hydroxylase; S-WAR7-HD, S-warfarin 7-hydroxylase; DEXO-DMT, Dextromethorphan O-demethylase.

The inhibition of CYP2B6 by CBD is attributed to the influence of free hydroxyl phenol groups and the pentyl side chain (Yamaori et al., 2011b). THC, CBD, and CBN are also capable of inhibiting CYP2D6, with CBD being the most potent competitive inhibitor. The possibility of structural influence by the hydroxyl groups and pentyl side chains was again discussed due to similar effects caused by the by-product of cannabinoid synthesis in *Cannabis sativa*, olivetol, which retains the hydroxyl groups and pentyl side chain and cannabidivarin (CBDV). In 2012, Yamaori and colleagues described a strong inhibition of the CYP2C9 isoform by THC, CBD, and CBN (Yamaori et al., 2012).

The same authors also evaluated mechanism-based inhibition (Yamaori et al., 2010; Yamaori et al., 2011a; Yamaori et al., 2011b; Yamaori et al., 2012). Mechanism-based inhibition of the CYP1A1 isoform was observed for THC, CBD, and CBN. CYP1A2 and CYP1B1 were inhibited by CBD (Yamaori et al., 2010). This inhibition is supported by a decrease in the IC_50_ value after a 20-min pre-incubation. For instance, the IC_50_ for CYP1A1 decreases from 0.411 μM at 0 min to 0.0767 µM after a 20-min pre-incubation with CBD. This pattern, although strongest for CBD, is observed for THC and CBN regarding the CYP1A1 isoform. Mechanism-dependent inhibition was also pointed out for CYP2A6 due to a marked decrease in IC_50_ for the various cannabinoids but not for CYP2B6, where the IC_50_ was similar (Yamaori et al., 2011a).

More recently, in 2020, Bansal and colleagues (Bansal et al., 2020) confirmed the inhibition of CYP1A2, 2C9, 2C19, 2D6, and 3A by THC and CBD (see Table 2). However, these recent studies suggest that the inhibition potencies of THC and CBD may have been underestimated due to methodological limitations, such as inadequate consideration of aqueous solubility or binding of cannabinoids to labware. The study used low-binding microcentrifuge tubes and included 0.2% bovine serum albumin in the reaction mixture to prevent underestimating the inhibition potencies. Thus, the IC_50_ and K_i_ values for the isoforms were much lower than those reported by studies of Yamaori and colleagues (Yamaori et al., 2010; Yamaori et al., 2011a; Yamaori et al., 2011b; Yamaori et al., 2012).

**TABLE 2:** Comparison of the IC_50_ (µM) values for inhibition studies of Yamaori et al, Bansal et al and Doohan et al. (Yamaori et al., 2010; Yamaori et al., 2011a; Yamaori et al., 2011b; Yamaori et al., 2012; Bansal et al., 2020; Doohan et al., 2021).

	CYP2C9			CYP2D6		
THC	1.02	ND	0.01	17.10	ND	1.28
CBD	2.70	2.5	0.17	6.01	ND	0.95
CBG	ND	1.0	ND	ND	ND	ND

IC_50_: half-maximal inhibitory concentration; Red: Yamaori and colleagues; Orange: Doohan and colleagues; Blue: Bansal and colleagues.

Note: The study of Bansal et al. used human liver microsomes, Yamaori et al. used recombinant CYPs, and Doohan et al. Used Supersomes™ containing P450 isoform.

Bansal and colleagues (Bansal et al., 2020) also investigated inhibitory potencies of THC metabolites, specifically 11-COOH-THC and 11-OH-THC. 11-OH-THC was found to be a reversible inhibitor of CYP2C9, 2C19, 2D6, and CYP3A, while 11-COOH-THC did not appear to have any inhibitory action. The clinical relevancy of inhibitory potencies of these metabolites is questionable since they have higher IC_50_ values than THC for the evaluated P450. Protein binding was again found to play an important role in the strength of time-dependent inhibition of CYP1A2 by CBD. In 2022, Bansal and colleagues (Bansal et al., 2022) expanded previous findings (Bansal et al., 2020) by determining time-dependent inhibition of CBD and its metabolites (7-OH-CBD and 7-COOH-CBD) for CYP2A6, CYP2B6, and CYP2C8 in human liver microsomes. They found that CBD, THC, and their metabolites are potent inhibitors of CYP2B6 and CYP2C8, but no time-dependent inhibition was observed for these P450 isoforms.

In 2020, Nagao and colleagues (Nagao et al., 2020) demonstrated that CBD has a non-linear pharmacokinetic profile due to the saturation of P450 enzyme activities and that it strongly inhibits CYP3A, similar to ketoconazole, in rats. Using a 13C-erythromycin breath test, they confirmed that CBD inhibits the metabolism of erythromycin, a CYP3A substrate, at doses of 10 mg/kg and 50 mg/kg.

In 2021, Doohan and colleagues (Doohan et al., 2021) explored the interaction of twelve cannabinoids with P450 isoforms. For this experiment, Supersomes™ was used, which represents a combination of a single recombinant P450 isoform and NADPH reductase expressed in insect cells. CBN and CBD were found to be inhibitors of caffeine metabolism by CYP1A2 and bupropion metabolism by CYP2B6, respectively, while having weak or no inhibitory effects on triazolam, nifedipine, and testosterone metabolism by CYP3A4 and dextromethorphan metabolism by CYP2D6. Most of cannabinoids inhibited tolbutamide metabolism by CYP2C9, with CBDA (cannabidiolic acid) having a stronger inhibitory effect than sulfaphenazole (Doohan et al., 2021).

Previous studies have shown that cannabinoids can inhibit various P450 isoforms, but there is evidence that they may also induce P450 enzymes. THC was found to induce CYP1A1 in HEPA-1 cells through direct action on the AhR, increasing messenger RNA (mRNA) levels and seven-ethoxyresorufin-o-deethylase (EROD) activity. Interestingly, although marijuana tar induced CYP1A1, the presence of THC in the tar seemed to restrict this induction compared to tobacco tar. However, at the transcriptional level, THC increased CYP1A1 mRNA (Roth et al., 2001). This suggests that THC may act in two ways: as a competitive inhibitor of CYP1A1, reducing the efficiency of binding by other substrates, and as an inducer of CYP1A1 mRNA. The structure of THC is comparable to the prototypical activator of AhR, 2,3,7,8-tetrachlorodibenzo-p-dioxin (TCDD), which can help explain the activation (although weak) of AhR and mild induction of CYP1A1 (Dauchy et al., 2009). These results were confirmed and expanded upon later when the major phytocannabinoids (THC, CBD, and CBN) were evaluated for their ability to induce CYP1A1 in human HepG2 cells (Yamaori et al., 2015). CBD was revealed to be the most potent inducer of them. The induction of CYP1A1 by CBD occurs via ligand-independent mechanisms through the activation of protein tyrosine kinase and phosphorylation of AhR, much like what happens with omeprazole (Daujat et al., 1992). It’s relevant to mention that crucial for this induction is the pentylresorcinol moiety of CBD (Yamaori et al., 2015). The increase in mRNA expression for CYP1A1 by CBD was independent of the activation of CB1, CB2, and TRPV1 receptors; hence, through docking analysis, the possibility of CBD interacting with the AhR-ARNT complex was put forward (Jang et al., 2023). This differs from the ligand-independent mechanisms mentioned before (Daujat et al., 1992; Yamaori et al., 2015).

While our primary focus was on THC and CBD, a growing body of research has expanded to include additional cannabinoids such as CBG. Doohan and colleagues identified CBG as a strong inhibitor of CYP2C9 metabolic activity (Doohan et al., 2021). Using human liver microsomes, extracts derived from various plant chemotypes with distinct cannabinoid profiles were evaluated for their ability to inhibit cytochrome P450 enzymes. Notably, extracts with high concentrations of CBG demonstrated strong inhibitory effects on CYP2C9 and CYP3A4 isoforms, comparable to those observed with CBD (Treyer et al., 2023). These findings align with those of Roy and colleagues (Roy et al., 2022), who reported CBG as a moderate inhibitor of CYP3A4 and—importantly—of CYP2D6, an interaction not previously documented. In contrast, other cannabinoids like CBC (cannabichromene) and CBDA need to be studied for potential drug-drug interactions.

In the essential oil of *Cannabis sativa*, there are also present other active ingredients, such as sesquiterpenes β-caryophyllene and trans-nerolidol. In recent years, multiple studies have been conducted to demonstrate their influence on the P450 enzyme system (Nguyen et al., 2017; Lněničková et al., 2018; Šadibolová et al., 2019) and the results are summarized in Table 3. Synergism between these compounds and cannabinoids has been previously suggested, for example, between β-caryophyllene and CBD (Blanton et al., 2022). β-caryophyllene is considered an CB_2_ agonist (Gertsch et al., 2008). CBD interacts with multiple G protein-coupled receptors including: TRPV1 (Bisogno et al., 2001) and 5-HT_1A_ (Russo et al., 2005). Cross-talk has been suggested to contribute to synergic analgesic effect (Blanton et al., 2022).

**TABLE 3:** Modulation of cytochrome P450 enzymes via nuclear receptors by cannabinoids and sesquiterpenes.

Molecule involved	Nuclear receptor	P450 isoform
2-AG	ERRγ	↑ CYP2E1 (Kim et al., 2013)
ACEA	ERRγ	↑ CYP2E1 (Kim et al., 2013)
AM-251	ERRγ	↓ CYP2E1 (Kim et al., 2013)
Noladin ether	ERRγ	↑ CYP7A1 (Zhang et al., 2015)
THC	AhR	↑ CYP1A1 (Roth et al., 2001; Dauchy et al., 2009)
β-caryophyllene*	RXR	↓↓ CYP3A (Nguyen et al., 2017)
β-caryophyllene*	RXR	↔ CYP3A4, CYP2C (Šadibolová et al., 2019)
Trans-nerolidol*	RXR	↔ CYP3A4, CYP2C (Šadibolová et al., 2019)
Trans-nerolidol*	RXR	↑ CYP2B, CYP3A and CYP2C (Lněničková et al., 2018)

* sesquiterpenes present in the essential oil of Cannabis sativa.

Kim and colleagues studied the influence of endocannabinoids on CYP2E1 (Kim et al., 2013). Authors conclude that mice exposed to alcohol also had high levels of 2-AG, and through its agonism on the CB_1_ receptors in hepatocytes, 2-AG increased the expression of ERRγ. This nuclear receptor further leads to increased expression of the CYP2E1 enzyme (see Table 3). These findings were confirmed using ACEA (CB_1_ agonist) and AM251 (CB_1_ antagonist), in which the latter blocked 2-AG signaling and diminished CYP2E1 induction (Kim et al., 2013). The inverse agonist of ERRγ, GSK5182, decreases CYP2E1 induction. Noladin ether, also known as 2-AGE (2-arachidonyl glyceryl ether), a cannabinoid-like compound, has been shown to induce CYP7A1 through the activation of ERRγ, which results from the prior activation of hepatic CB_1_ receptors. Although this isoform is not directly related to drug metabolism, it is associated with liver dysfunction due to the accumulation of bile acids, which could disrupt enterohepatic recirculation and, therefore, indirectly affect drug metabolism (Zhang et al., 2015). Recently, in a study about the role of cannabinoids in protection against retina degeneration, it was observed that the intravitreal injection of ACEA, a selective CB_1_ receptor agonist, and AM251, a selective CB_1_ antagonist, interfere with the transcription of CYP1A1. While ACEA led to decreased AhR expression, AM251 led to the opposite and, thus, CYP1A1 induction (Soliño et al., 2022).

### 3.3 Endocannabinoid system and modulation of neurotransmitter systems

The importance of different neurotransmitter systems in the regulation of P450 enzymes has been previously described (Wójcikowski and Daniel, 2011; Kuban and Daniel, 2021; Haduch et al., 2022; 2023; Pukło et al., 2023). Endocannabinoid system is a retrograde regulator of the other neurotransmitter systems, particularly CB_1_ receptors are predominantly localized on presynaptic neuronal terminals, where they regulate the release of various neurotransmitters (Marsicano and Lutz, 1999; Schlicker and Kathmann, 2001). Endocannabinoids are synthesized and released by postsynaptic neurons and act retrogradely on presynaptic CB_1_ receptors to inhibit the release of GABA, glutamate, acetylcholine, serotonin, and noradrenaline Additional complexity arises from the ability of CB_1_ receptors to form heteromers with other G-protein coupled receptors (Hermann et al., 2002; Carriba et al., 2007; Chen et al., 2016). Interactions between the endocannabinoid and dopaminergic systems may also occur indirectly via GABAergic (Sperlágh et al., 2009) and glutamatergic pathways (Melis et al., 2004). Given that the endocannabinoid system is closely linked with these systems, it is possible to hypothesize that it influences drug metabolism by modulating the expression of P450 enzymes (Zendulka et al., 2016).

**FIGURE 1:**
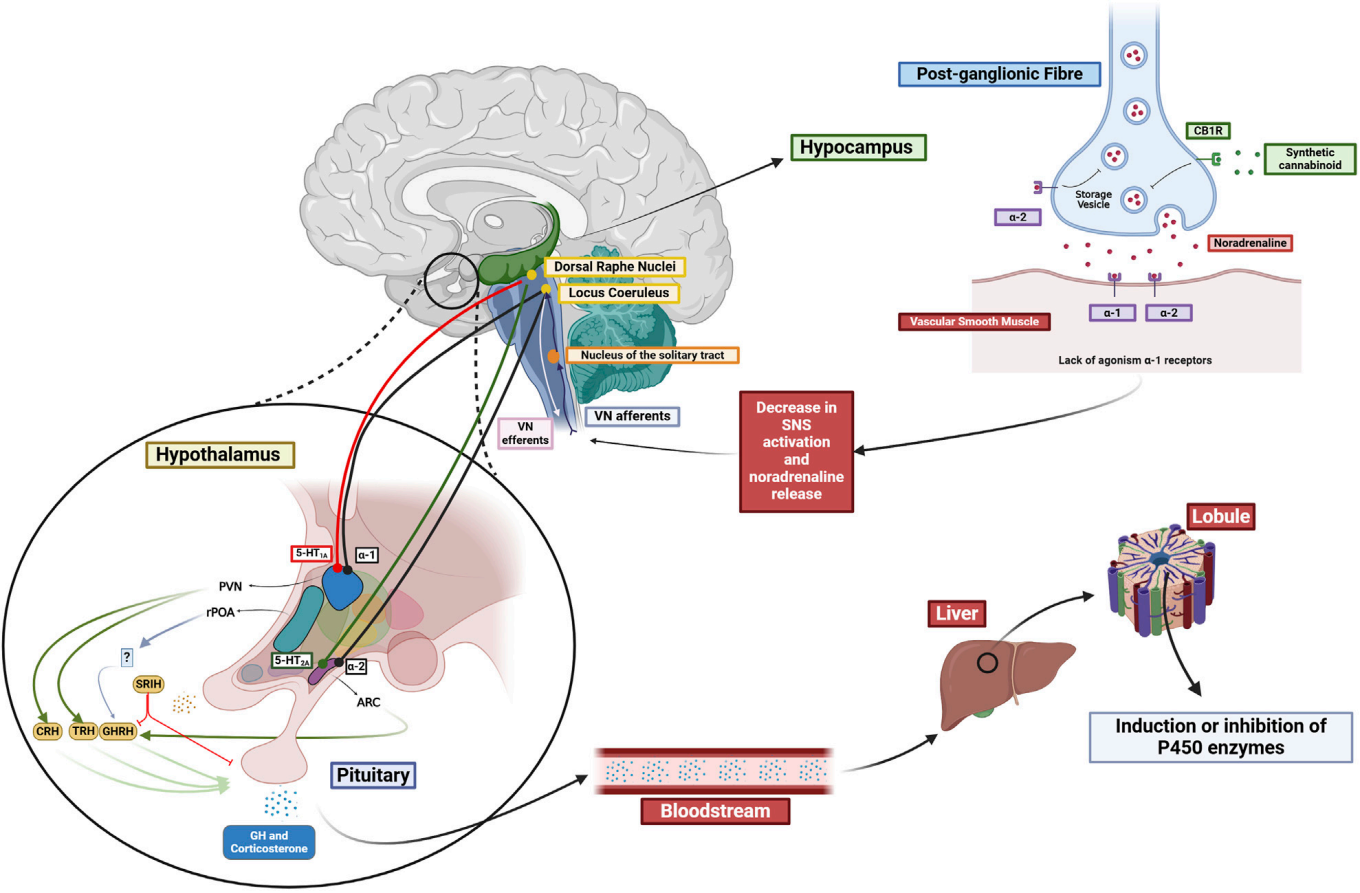
Proposed influence of the different neurotransmitter systems in the regulation of P450 enzymes. Cannabinoids interaction with CB1 receptors influences the modulation of various neurotransmitter systems (NA, DA, 5-HT). Possible hypothalamic influence in the PVN and ARC interfering with the release of several hormones, which, in turn, interact with nuclear receptors (PPARα, GR, PXR, and CAR), thereby affecting the inhibition or induction of P450 enzymes. Created in BioRender (https://BioRender.com/j18d041).

Neurotransmitters such as noradrenaline, dopamine, serotonin, and glutamate are indirectly involved in regulating the P450 enzymes, and cannabinoid signaling affects these neurotransmitters. This action on the P450 protein levels and activity is mediated by the hypothalamic-pituitary axis, which can stimulate or suppress hormone release, including growth hormone, corticosterone, and thyroid hormones. The hypothalamic paraventricular (PVN) and arcuate nucleus (ARC) are crucial in this process. The PVN contains neuroendocrine neurons that produce thyrotropin-releasing hormone (TRH), corticotropin-releasing hormone (CRH), and somatostatin (SRIH). The ARC is responsible for the production of growth hormone-releasing hormone (GHRH), which leads to the production of growth hormone (GH) by the pituitary (see Figure 1).

Since 2007, Daniel and colleagues (Wójcikowski et al., 2007; 2008; Wójcikowski and Daniel, 2008; 2009) have demonstrated the relation between the brain dopaminergic system and the regulation of P450 enzymes. Using 6-hydroxydopamine (6-OH-DA), they caused dopaminergic pathways (tuberoinfundibular, mesolimbic, nigrostriatal) lesions. The lesion of the tuberoinfundibular pathway resulted in the inhibition of CYP2B, CYP3A and CYP2C6. The lesion of the mesolimbic pathway resulted in modest induction of CYP1A. The lack of a neuronal connection between the tuberoinfundibular pathway and the pituitary gland, and consequently the lack of endogenous hormone release, explains some of the unchanged P450 activities. Nuclear receptors (GR, PXR, CAR, PPARα) are responsible for the regulation of P450 activity. PPARα seems to be activated by thyroid hormones, leading to an induction of CYP1A. Functional crosstalk between GR, PXR and CAR seems to regulate the activation of CYP2B and CYP3A (see Table 4).

**TABLE 4:** Impact of 6-OH-DA and D_2_ agonists on the P450 activity.

	Tuberoinfundibular pathway	Mesolimbic pathway
6-OH-DA		
CYP1A	ND	↑
CYP2A	ND	ND
CYP2B	↓	ND
CYP2C6	↓	ND
CYP2C11	ND	ND
CY3A	↓	ND
D2 agonists		
CYP1A	↓	↓
CYP2A	ND	ND
CYP2B	↑	ND
CYP2C6	ND	ND
CYP2C11	↑	ND
CY3A	↑	↑

6-OH-DA, 6-hydroxy-dopamine; ND, no change in P450 activity.

Complementing previous findings activation of D_2_ receptors (e.g., apomorphine, amphetamine, quinpirole and SKF82958) led to an increase in GH and a decrease in triiodothyronine (T_3_) in the pituitary, which led to induction of CYP2B, CYP2C11, and CYP3A and inhibition of CYP1A. D_2_ receptor activation in the *nucleus accumbens* increased corticosterone and decreased T_3,_ causing inhibition of CYP1A activity and induction of CYP3A (see Table 4) (Wójcikowski et al., 2008).

The researchers also investigated the interaction of neurotransmitter systems and hypothalamic/pituitary hormones through the pharmacological depletion of the respective neurotransmitters (Kot and Daniel, 2011). N - (2 - chloroethyl) - N -ethyl- 2 - bromobenzylamine (DSP-4) is an inhibitor of the reuptake of noradrenaline (Ross et al., 1973) and leads to a diminution of dopamine-β-hydroxylase activity (Ross, 1976), therefore functioning as a noradrenergic toxin. Similarly, p-chloroamphetamine (PCA) act as an inhibitor of serotonin uptake and an inhibitor of tryptophan hydroxylase (Sanders-Bush et al., 1975). The p-chlorophenylalanine (PCPA) inhibits tryptophan hydroxylase, therefore acting as a serotonergic toxin (Jéquier et al., 1967) (see Table 5). To expand on these previous results, a tryptophan-free diet was used as an alternative approach to test the influence of serotonin on the P450 enzyme system (see Table 6) (Kot et al., 2012). These experiments revealed that increased hypothalamic serotonin levels led to increased activity of the various CYP1A, CYP2A, CYP2B, CYP2C6, and CYP3A isoforms and decreased activity of CYP2C11. The deviation for CYP2A and CYP2B when comparing the tryptophan-free diet after 3 and 7 days is due to increased serotonin levels in the plasma and a plateau in the brain, which might be a consequence of an induced release of serotonin from the platelets acting as a reservoir for serotonin. This might be explained through the activation of 5-HT_1A_, (hypothalamic) 5-HT_2A_, 5-HT_2B_ (pituitary) and 5-HT_2C_ (adrenal cortex) receptors which increase adrenocorticotropic hormone (ACTH), GH and corticosterone levels (see Figure 1). After 3 weeks, a drop in serotonin, dopamine and respective metabolites was observed in the CNS and plasma. As a result, CYP1A, CYP2A, CYP2B, CYP2C6 and CYP3A had increased activity while the opposite was true for CYP2C11.

**TABLE 5:** Influence of noradrenergic and serotonergic toxins on P450 activity.

System	Noradrenergic and serotonergic		
Substance	PCP	PCPA	DSP-4
Activity			
CYP1A	↑	↑	ND
CYP2A	ND	ND	ND
CYP2B	ND	ND	↓
CYP2C6	ND	ND	ND
CYP2C11	↓	↓	↓
CY3A	↓	↓	↓

PCP, p-chlorophenylalanine; PCPA, p-chloroamphetamine; DSP-4, N-(2-chloroethyl)-N-ethyl-2-bromobenzylamine; ND, no change in P450 activity.

**TABLE 6:** Influence of tryptophan-free diet on P450 activity.

System	Serotonergic		
Substance	TFD 3 Days	TFD 7 days	TFD 21 days
Activity			
CYP1A	ND	ND	↑
CYP2A	ND	↑	↑
CYP2B	↑	↓	↑
CYP2C6	↑	↑	↑
CYP2C11	↓	↓	↓
CY3A	↑	↑	↑

TFD, tryptophan-free diet; ND, no change in P450 activity.

The intracerebral administration of DSP-4, a noradrenergic toxin, and 5,7-dihydroxytryptamine (5,7-DHT), a serotonergic toxin, changed P450 enzyme activity (see Table 7). These changes seem to be directly associated with changes in growth hormone levels (Bromek et al., 2013; Rysz et al., 2016).

**TABLE 7:** Influence of DSP-4 and 5,7-DHT on hormones and P450 activity.

	Noradrenergic toxin		Serotonergic toxin	
Substance	DSP-4		5,7-DHT	
Lesion	PVN	ARC	PVN	ARC
Hormonal Levels	↓Somatostatin → ↑GH	↓ GHRH → ↓GH	↑GH and ↑ Testosterone	↓GH
Activity				
CYP1A	ND	↓	ND	ND
CYP2A	↑	ND	ND	ND
CYP2B	ND	ND	ND	ND
CYP2C6	ND	ND	ND	ND
CYP2C11	↑	↓	↑	↓
CY3A	↑	↓	ND	ND

DSP-4, N - (2 - chloroethyl) - N -ethyl- 2, bromobenzylamine; 5,7-DHT, 5,7-Dihydroxytryptamine; PVN, paraventricular nucleus; ARC, arcuate nucleus; GHRH, growth hormone-releasing hormone; GH, growth hormone; ND, no change in P450 activity.

More recently, in 2018, Bromek et al. identified that 5-HT_1A_ receptors in PVN play a crucial role in the serotonergic regulation of P450 enzymes (Bromek et al., 2018). The agonist of the 5-HT_1A_ receptor led to an increase in somatostatin, which in turn suppresses growth hormone release and, consequently, decreases CYP2C11 and CYP3A expression and enzyme activity. A decrease in corticotropin-releasing (CRT) was also observed. In 2019, Bromek and colleagues (Bromek et al., 2019), using the same experimental design, proved the importance of 5-HT_2_ in the regulation of P450. Both single and repeated administration of a selective 5-HT_2_ agonist 1-[2,5-dimethoxy-4-iodophenyl]-2-aminopropan increased CYP2C11 and CYP3A activity in the liver, resulting from increased synthesis of GHRH and, consequently, GH.

Considering the results of the previous studies, it is possible to outline a hypothesis about the involvement of cannabinoids in the HPA axis and subsequent regulation of the P450 enzymes (see Figure 1 below). Since CBD is known as an agonist of 5-HT_1A_ (Russo et al., 2005), it would technically mediate similar effects as 5-carboxyamidotryptamine (5-CT) and 8-OH-DPAT (a 5HT_1_ serotonergic agonists) in the previous experiment (Bromek et al., 2018). Besides the direct interactions of cannabinoid ligands with DA, 5-HT or adrenergic receptors, endocannabinoids act as retrograde regulators of the above-mentioned systems (see Figure 1).

By this, various homotropic and heterotropic neuronal inter-regulations exist (Hermann et al., 2002; Gonzalez et al., 2009; Sperlágh et al., 2009). It has been hypothesised that at least a part of the effects of cannabinoids is regulated via dopamine (Di Marzo, 2009; Di Giovanni, 2010; Fernández-Ruiz et al., 2010; Kuepper et al., 2010; El Khoury et al., 2012). It is known that CB_1_ receptor stimulation increases dopamine release in the *nucleus accumbens* (Sperlágh et al., 2009). CB_1_ receptors are, similarly to α_1_ receptors, present on presynaptic elements in the *nucleus accumbens*, an integral part of the mesolimbic neuronal pathway (Chevaleyre et al., 2006; Mitrano et al., 2012). By this, *nucleus accumbens* also receives 5-HT signalling (Brown and Molliver, 2000).

In light of published papers (Sperlágh et al., 2009; Di Giovanni, 2010; Fernández-Ruiz et al., 2010; Kuepper et al., 2010; El Khoury et al., 2012), the overall effects of CB ligands could also be mediated via the cross-talk between endocannabinoid system and above-mentioned hormones, neuroregulatory pathways and subsequently influence P450 metabolic activity.

Another possible regulatory pathway of P450 by cannabinoids involves indirect influence on the noradrenergic and serotonergic transmission in specific brain regions, such as the *locus coeruleus* (major noradrenergic brainstem nucleus) and the *dorsal raphe nuclei* (the largest serotonergic nucleus) (Michelsen et al., 2008; Breton-Provencher et al., 2021). The influence on these sites could potentially have more significant consequences in the hypothalamus, affecting P450 expression.

Synthetic cannabinoids WIN 55212-2, and CP55940 increase noradrenergic activity *in vivo* (Mendiguren and Pineda, 2006). This effect does not occur locally by acting on CB_1_ receptors in the *locus coeruleus.* Instead, it is mediated through the systemic administration of CB_1_ agonists acting on peripheral CB_1_ receptors, resulting in hypotension due to the inhibition of noradrenaline release from postganglionic sympathetic neurons (Niederhoffer et al., 2003). The resulting hypotension leads to the activation of the neurons in the *locus coeruleus* through the vagus nerve (see Figure 1).

Regarding glutamatergic neurotransmission, activation of CB_1_ receptors by anandamide and synthetic cannabinoids WIN 55212-2 and CP55940 increase glutamatergic signaling to the PVN and ARC by activating NMDA receptors in *locus coeruleus* (Mendiguren and Pineda, 2004). This is important because *locus coeruleus* has innervations to the PVN and ARC in the hypothalamus (Szabadi, 2013). If an increase in the *locus coeruleus* activity were to occur, it could result in NA release and activation of parvocellular neurons in the PVN through α_1_-adrenoreceptors. These neurons are responsible for producing TRH, CRH, and SRIH. Similarly, if the ARC were to be activated through α_2_-adrenoreceptors consequently, it would lead to the release of growth hormone, which could subsequently interfere with the expression of specific P450 isoforms.

## 4 Future perspectives

The endocannabinoid system has been the focus of extensive research due to its promising therapeutic potential, with studies conducted in various fields, including psychiatry, inflammatory diseases and cancer (Groce, 2018). It is important to note that the function of the endocannabinoid system can be altered not only by cannabinoid receptor ligands but also indirectly by affecting the synthesis and degradation of endogenous cannabinoids. This offers a variety of pharmacological mechanisms for new drugs targeting the alteration of the activity of this important regulator of physiological and pathophysiological processes. Notwithstanding the elevated clinical expectations that pertain to the development of drugs based on the modulation of the endocannabinoid system, setbacks are also present (Di Marzo, 2018). It is also important to note the rising abuse of synthetic cannabinoids (Alzu’bi et al., 2024) and the legalization of cannabis for recreational use in many countries, both of which contribute to a growing population of cannabinoid users.

To better assess the interaction potential of existing or novel cannabinoids and other compounds affecting endocannabinoid system activity, it would be advisable to incorporate additional tests beyond the standard *in vitro* inhibition studies currently required by the EMA and FDA during preclinical drug evaluation. These supplementary assays could provide a more comprehensive understanding of potential effects on P450, including mechanisms beyond direct enzyme inhibition. Such tests should focus on the influence of studied drugs on the activation/inhibition of nuclear receptors regulating P450 hepatic activity, namely, CAR, AhR, and PXR. Because the endocannabinoid system is dynamic with many feedbacks and interplays with other neurotransmitters that change in time, the time factor should also be involved in the P450 testing. Different effects could be seen in both acute and chronic exposition to cannabinoids, similarly to modulation of glutamatergic neurotransmission by cannabis (Chowdhury et al., 2024).

## 5 Conclusion

This review addresses the critical knowledge gap regarding how cannabinoids interact with cytochrome P450 enzymes. As global cannabis use continues to rise, particularly among older adults, and with increasing THC concentrations in cannabis products (Manthey et al., 2021), understanding these interactions is essential for ensuring safe and effective use. This significance is further highlighted by the fact that older age groups are often polymedicated. While some studies highlight the inhibition of various P450 isoforms by THC, CBD, CBN, and CBDA, few explain the underlying mechanisms. Based on the existing literature, we hypothesize how the endocannabinoid system interacts with the monoaminergic and glutamatergic systems, its impact on the HPA axis, and how these interactions may ultimately influence P450 enzyme expression. Phytocannabinoids or synthetic cannabinoids are capable of direct drug-drug interactions at the level of P450 enzymes, as well as possibly capable of triggering a change in 5-HT, DA or NA signaling, which, in turn, might influence the liver P450 activity via hormones and nuclear receptors. Since the studies that have been published so far have not investigated the “net” central contribution of cannabinoid ligands to the “overall” change in the liver P450 activity, this hypothesis needs to be proved or disproved by well-designed experiments. This narrative review compels to provide further insights and to motivate research in this understudied topic.
